# 2-[3-(4-Bromo­phenyl)-5-(4-fluoro­phenyl)-4,5-di­hydro-1*H*-pyrazol-1-yl]-4-phenyl-1,3-thia­zole

**DOI:** 10.1107/S1600536813010039

**Published:** 2013-04-17

**Authors:** Bakr F. Abdel-Wahab, Hanan A. Mohamed, Seik Weng Ng, Edward R. T. Tiekink

**Affiliations:** a Applied Organic Chemistry Department, National Research Centre, Dokki, 12622 Giza, Egypt; b Department of Chemistry, University of Malaya, 50603 Kuala Lumpur, Malaysia; c Chemistry Department, Faculty of Science, King Abdulaziz University, PO Box 80203 Jeddah, Saudi Arabia

## Abstract

In the title compound, C_24_H_17_BrFN_3_S, the pyrazole ring is almost planar (r.m.s. deviation = 0.043 Å), with all but the perpendicular fluoro­benzene ring substituents [dihedral angle = 77.9 (3)°] being very approximately coplanar [dihedral angle with the 2-thienyl ring = 19.4 (3)° and with the bromo­benzene ring = 20.3 (3)°; dihedral angle between the 2-thienyl and attached phenyl ring = 11.0 (4)°], so that the mol­ecule has a T-shape. In the crystal, supra­molecular chains along the *b-*axis direction are sustained by C—H⋯S and C—Br⋯π inter­actions.

## Related literature

For the biological activities and synthesis of pyrazolin-1-carbo­thio­amides, see: Abdel-Wahab *et al.* (2012 ▶); Lv *et al.* (2011 ▶). For a related structure, see: Abdel-Wahab *et al.* (2013 ▶).
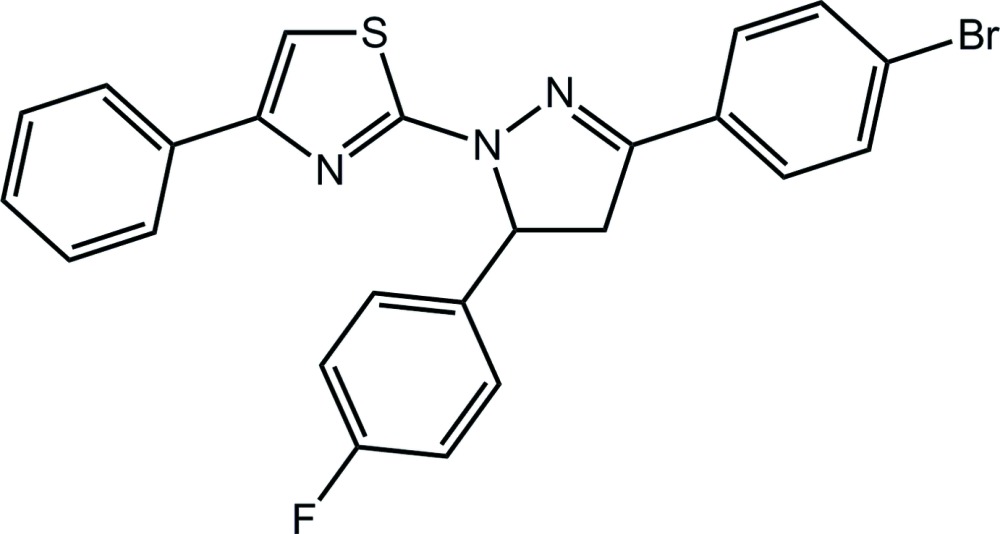


## Experimental

### 

#### Crystal data

C_24_H_17_BrFN_3_S
*M* *_r_* = 478.38
Monoclinic, 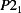

*a* = 13.747 (2) Å
*b* = 5.6695 (13) Å
*c* = 14.280 (3) Å
β = 106.94 (2)°
*V* = 1064.7 (4) Å^3^
*Z* = 2
Mo *K*α radiation
μ = 2.05 mm^−1^
*T* = 295 K
0.30 × 0.10 × 0.02 mm

#### Data collection

Agilent SuperNova Dual diffractometer with an Atlas detector
Absorption correction: multi-scan (*CrysAlis PRO*; Agilent, 2011 ▶) *T*_min_ = 0.937, *T*_max_ = 1.000
7430 measured reflections
4124 independent reflections
1947 reflections with *I* > 2σ(*I*)
*R*_int_ = 0.052

#### Refinement

*R*[*F* ^2^ > 2σ(*F* ^2^)] = 0.052
*wR*(*F* ^2^) = 0.136
*S* = 0.95
4124 reflections
271 parameters
1 restraint
H-atom parameters constrained
Δρ_max_ = 0.26 e Å^−3^
Δρ_min_ = −0.32 e Å^−3^
Absolute structure: Flack (1983 ▶), 1440 Friedel pairs
Flack parameter: −0.022 (15)

### 

Data collection: *CrysAlis PRO* (Agilent, 2011 ▶); cell refinement: *CrysAlis PRO*; data reduction: *CrysAlis PRO*; program(s) used to solve structure: *SHELXS97* (Sheldrick, 2008 ▶); program(s) used to refine structure: *SHELXL97* (Sheldrick, 2008 ▶); molecular graphics: *ORTEP-3 for Windows* (Farrugia, 2012 ▶) and *DIAMOND* (Brandenburg, 2006 ▶); software used to prepare material for publication: *publCIF* (Westrip, 2010 ▶).

## Supplementary Material

Crystal structure: contains datablock(s) global, I. DOI: 10.1107/S1600536813010039/qm2094sup1.cif

Structure factors: contains datablock(s) I. DOI: 10.1107/S1600536813010039/qm2094Isup2.hkl

Supplementary material file. DOI: 10.1107/S1600536813010039/qm2094Isup3.cml

Additional supplementary materials:  crystallographic information; 3D view; checkCIF report

## Figures and Tables

**Table 1:** Hydrogen-bond geometry (Å, °) *Cg*1 is the centroid of the C13–C18 benzene ring.

*D*—H⋯*A*	*D*—H	H⋯*A*	*D*⋯*A*	*D*—H⋯*A*
C4—H4⋯S1^i^	0.98	2.84	3.734 (7)	153
C22—Br1⋯*Cg*1^ii^	1.897 (6)	3.644 (3)	5.265 (7)	141.6 (3)

Symmetry codes: (i) 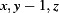; (ii) 
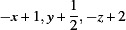
.

**Fig. 1.**
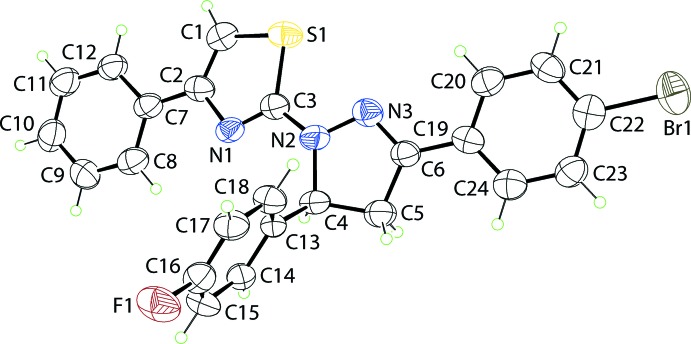
The molecular structure of (I) showing the atom-labelling scheme and displacement ellipsoids at the 35% probability level.

**Fig. 2.**
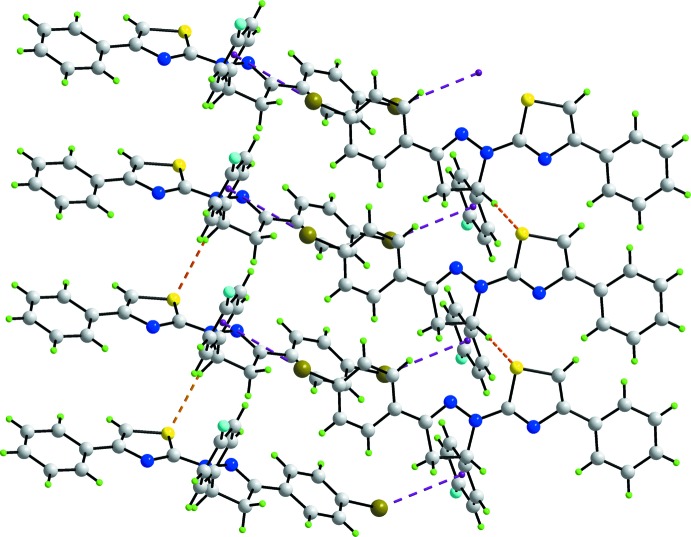
A view of the supramolecular chain along the b axis in (I) sustained by C—H···S and C—Br···.π interactions, shown as orange and purple dashed lines, respectively.

**Fig. 3.**
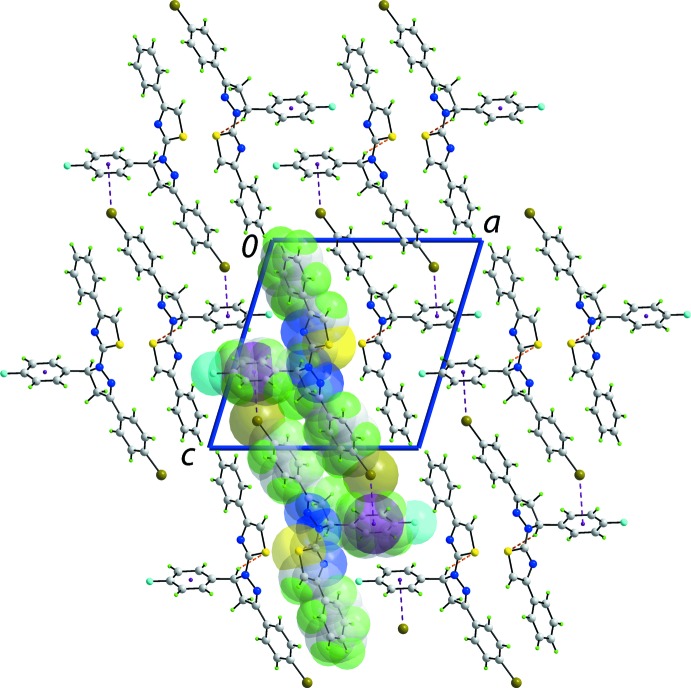
A view of the crystal packing in projection down the b axis. One supramolecular chain has been highlighted in space-filling mode. The C—H···S and C—Br···.π interactions are shown as orange and purple dashed lines, respectively.

## supplementary crystallographic information

### Comment

Pyrazolin-1-carbothioamide derivatives are known to possess biological activity (Abdel-Wahab *et al.*, 2012; Lv *et al.*, 2011) and in connection of on-going studies in this area, the title compound(I) was characterized.

In (I), the pyrazolyl ring is planar with a r.m.s. deviation of 0.043 Å; maximum deviations: 0.035 (7) Å [C5] and -0.034 (6) Å [C4]. The adjacent 2-thienyl ring is inclined [dihedral angle = 19.4 (3)°] as is the bromo-benzene ring [dihedral angle = 20.3 (3)°] but the fluoro-benzene ring is approximately perpendicular [77.9 (3)°]. Finally, a twist exists between the 2-thienyl and attached phenyl ring [11.0 (4)°]. The structure resembles the T-shapes observed for the two independent molecules of the recently determined closely related derivative where the bromo-benzene substituent in (I) is now a *p*-tolyl group (Abdel-Wahab *et al.*, 2013).

Supramolecular chains along the *b* axis are formed in the crystal packing by C—H···S and C—Br···π interactions, Fig. 2 and Table 1. These stack in the crystal structure with no specific interactions between them, Fig. 3.

### Experimental

The title compound was prepared according to the reported method (Lv *et al.*, 2011). Yellow crystals were obtained from its ethanol solution by slow evaporation at room temperature.

### Refinement

Carbon-bound H-atoms were placed in calculated positions (C—H = 0.93 to 0.98 Å) and were included in the refinement in the riding model approximation, with *U*_iso_(H) = 1.2*U*_equiv_(C).

### Crystal data

**Table:**

C_24_H_17_BrFN_3_S	*F*(000) = 484
*M**_r_* = 478.38	*D*_x_ = 1.492 Mg m^−^^3^
Monoclinic, *P*2_1_	Mo *K*α radiation, λ = 0.71073 Å
Hall symbol: P 2yb	Cell parameters from 1208 reflections
*a* = 13.747 (2) Å	θ = 2.9–27.5°
*b* = 5.6695 (13) Å	µ = 2.05 mm^−^^1^
*c* = 14.280 (3) Å	*T* = 295 K
β = 106.94 (2)°	Plate, yellow
*V* = 1064.7 (4) Å^3^	0.30 × 0.10 × 0.02 mm
*Z* = 2	

### Data collection

**Table:**

Agilent SuperNova Dual diffractometer with an Atlas detector	4124 independent reflections
Radiation source: SuperNova (Mo) X-ray Source	1947 reflections with *I* > 2σ(*I*)
Mirror monochromator	*R*_int_ = 0.052
Detector resolution: 10.4041 pixels mm^-1^	θ_max_ = 27.6°, θ_min_ = 2.9°
ω scan	*h* = −17→17
Absorption correction: multi-scan (*CrysAlis PRO*; Agilent, 2011)	*k* = −7→7
*T*_min_ = 0.937, *T*_max_ = 1.000	*l* = −17→18
7430 measured reflections	

### Refinement

**Table:**

Refinement on *F*^2^	Secondary atom site location: difference Fourier map
Least-squares matrix: full	Hydrogen site location: inferred from neighbouring sites
*R*[*F*^2^ > 2σ(*F*^2^)] = 0.052	H-atom parameters constrained
*wR*(*F*^2^) = 0.136	*w* = 1/[σ^2^(*F*_o_^2^) + (0.042*P*)^2^] where *P* = (*F*_o_^2^ + 2*F*_c_^2^)/3
*S* = 0.95	(Δ/σ)_max_ < 0.001
4124 reflections	Δρ_max_ = 0.26 e Å^−^^3^
271 parameters	Δρ_min_ = −0.32 e Å^−^^3^
1 restraint	Absolute structure: Flack (1983), 1440 Friedel pairs
Primary atom site location: structure-invariant direct methods	Flack parameter: −0.022 (15)

### Special details

**Table:**

Geometry. All e.s.d.'s (except the e.s.d. in the dihedral angle between two l.s. planes) are estimated using the full covariance matrix. The cell e.s.d.'s are taken into account individually in the estimation of e.s.d.'s in distances, angles and torsion angles; correlations between e.s.d.'s in cell parameters are only used when they are defined by crystal symmetry. An approximate (isotropic) treatment of cell e.s.d.'s is used for estimating e.s.d.'s involving l.s. planes.
Refinement. Refinement of *F*^2^ against ALL reflections. The weighted *R*-factor *wR* and goodness of fit *S* are based on *F*^2^, conventional *R*-factors *R* are based on *F*, with *F* set to zero for negative *F*^2^. The threshold expression of *F*^2^ > σ(*F*^2^) is used only for calculating *R*-factors(gt) *etc*. and is not relevant to the choice of reflections for refinement. *R*-factors based on *F*^2^ are statistically about twice as large as those based on *F*, and *R*- factors based on ALL data will be even larger.

### Fractional atomic coordinates and isotropic or equivalent isotropic displacement parameters (Å^2^)

**Table:**

	*x*	*y*	*z*	*U*_iso_*/*U*_eq_	
Br1	0.80996 (5)	0.5031 (2)	1.12976 (5)	0.1040 (4)	
S1	0.42126 (13)	0.7866 (3)	0.50255 (13)	0.0748 (5)	
N1	0.2837 (3)	0.4642 (11)	0.4419 (4)	0.0630 (14)	
N3	0.4367 (3)	0.5425 (12)	0.6880 (4)	0.0660 (14)	
F1	−0.0906 (3)	0.3496 (8)	0.6421 (3)	0.1023 (14)	
N2	0.3571 (4)	0.4670 (11)	0.6112 (4)	0.0710 (15)	
C1	0.3593 (4)	0.7686 (14)	0.3793 (4)	0.0702 (18)	
H1	0.3715	0.8689	0.3324	0.084*	
C2	0.2906 (4)	0.5902 (12)	0.3589 (5)	0.0646 (18)	
C3	0.3476 (4)	0.5541 (12)	0.5189 (5)	0.0594 (16)	
C4	0.3109 (4)	0.2426 (12)	0.6306 (4)	0.0629 (17)	
H4	0.3147	0.1233	0.5822	0.075*	
C5	0.3835 (5)	0.1799 (13)	0.7310 (5)	0.075 (2)	
H5A	0.3465	0.1557	0.7786	0.090*	
H5B	0.4218	0.0383	0.7273	0.090*	
C6	0.4522 (4)	0.3888 (12)	0.7575 (5)	0.0614 (17)	
C7	0.2229 (4)	0.5194 (15)	0.2635 (4)	0.0657 (16)	
C8	0.1643 (5)	0.3151 (14)	0.2503 (5)	0.077 (2)	
H8	0.1693	0.2152	0.3032	0.092*	
C9	0.0991 (5)	0.2595 (18)	0.1599 (6)	0.090 (2)	
H9	0.0608	0.1220	0.1527	0.108*	
C10	0.0896 (6)	0.4009 (16)	0.0809 (6)	0.089 (3)	
H10	0.0448	0.3605	0.0205	0.107*	
C11	0.1458 (6)	0.6017 (16)	0.0904 (6)	0.086 (2)	
H11	0.1399	0.6990	0.0366	0.103*	
C12	0.2119 (5)	0.6601 (13)	0.1811 (5)	0.073 (2)	
H12	0.2501	0.7976	0.1870	0.088*	
C13	0.2020 (4)	0.2804 (12)	0.6295 (4)	0.0513 (14)	
C14	0.1304 (4)	0.1072 (11)	0.5906 (4)	0.0625 (17)	
H14	0.1488	−0.0251	0.5612	0.075*	
C15	0.0310 (5)	0.1304 (13)	0.5955 (5)	0.0724 (19)	
H15	−0.0176	0.0157	0.5691	0.087*	
C16	0.0072 (5)	0.3227 (15)	0.6393 (5)	0.0688 (19)	
C17	0.0750 (5)	0.4978 (15)	0.6783 (4)	0.0709 (16)	
H17	0.0552	0.6299	0.7067	0.085*	
C18	0.1738 (5)	0.4744 (15)	0.6745 (4)	0.0679 (17)	
H18	0.2217	0.5895	0.7023	0.081*	
C19	0.5332 (5)	0.4234 (12)	0.8500 (5)	0.0619 (18)	
C20	0.5968 (5)	0.6173 (13)	0.8638 (5)	0.074 (2)	
H20	0.5849	0.7331	0.8156	0.089*	
C21	0.6774 (5)	0.6428 (13)	0.9471 (5)	0.077 (2)	
H21	0.7196	0.7741	0.9548	0.092*	
C22	0.6949 (5)	0.4748 (17)	1.0181 (4)	0.0730 (19)	
C23	0.6317 (5)	0.2821 (15)	1.0095 (5)	0.076 (2)	
H23	0.6431	0.1703	1.0592	0.091*	
C24	0.5504 (5)	0.2581 (15)	0.9248 (5)	0.0751 (19)	
H24	0.5069	0.1295	0.9183	0.090*	

### Atomic displacement parameters (Å^2^)

**Table:**

	*U*^11^	*U*^22^	*U*^33^	*U*^12^	*U*^13^	*U*^23^
Br1	0.0919 (5)	0.1352 (8)	0.0810 (5)	0.0014 (6)	0.0188 (4)	−0.0233 (6)
S1	0.0741 (10)	0.0648 (12)	0.0979 (12)	−0.0075 (10)	0.0447 (10)	−0.0009 (10)
N1	0.052 (3)	0.063 (4)	0.081 (3)	0.003 (3)	0.031 (3)	0.019 (3)
N3	0.055 (3)	0.068 (4)	0.080 (3)	−0.002 (3)	0.028 (3)	0.002 (4)
F1	0.070 (2)	0.097 (3)	0.157 (4)	−0.005 (2)	0.060 (3)	−0.005 (3)
N2	0.065 (3)	0.068 (4)	0.081 (3)	−0.010 (3)	0.024 (3)	0.017 (3)
C1	0.076 (4)	0.067 (5)	0.079 (4)	0.009 (4)	0.040 (4)	0.006 (4)
C2	0.055 (3)	0.059 (5)	0.090 (5)	0.001 (3)	0.036 (4)	−0.001 (4)
C3	0.048 (3)	0.058 (5)	0.081 (4)	0.005 (3)	0.032 (3)	0.005 (4)
C4	0.068 (4)	0.048 (4)	0.077 (4)	−0.006 (3)	0.028 (4)	0.005 (3)
C5	0.063 (4)	0.063 (5)	0.100 (5)	0.000 (4)	0.027 (4)	0.013 (4)
C6	0.052 (4)	0.055 (4)	0.080 (4)	0.002 (3)	0.024 (3)	0.006 (4)
C7	0.060 (3)	0.063 (5)	0.083 (4)	0.005 (4)	0.035 (3)	0.014 (5)
C8	0.082 (4)	0.063 (5)	0.092 (5)	0.008 (4)	0.036 (4)	0.010 (4)
C9	0.072 (4)	0.100 (7)	0.094 (6)	−0.013 (5)	0.020 (4)	0.007 (5)
C10	0.073 (5)	0.107 (8)	0.087 (5)	0.013 (5)	0.024 (4)	0.023 (5)
C11	0.080 (5)	0.091 (7)	0.094 (6)	0.005 (5)	0.036 (5)	0.028 (5)
C12	0.070 (4)	0.067 (5)	0.088 (5)	0.001 (4)	0.031 (4)	0.016 (4)
C13	0.050 (3)	0.052 (4)	0.052 (3)	−0.007 (3)	0.014 (3)	0.007 (3)
C14	0.065 (4)	0.048 (4)	0.071 (4)	−0.001 (3)	0.015 (3)	0.001 (3)
C15	0.066 (4)	0.062 (5)	0.091 (5)	−0.022 (4)	0.026 (4)	−0.008 (4)
C16	0.066 (4)	0.074 (6)	0.074 (4)	0.007 (4)	0.032 (4)	0.010 (4)
C17	0.081 (4)	0.051 (4)	0.085 (4)	0.002 (5)	0.031 (4)	−0.007 (4)
C18	0.067 (4)	0.059 (5)	0.083 (4)	−0.004 (4)	0.029 (3)	−0.006 (4)
C19	0.056 (4)	0.056 (5)	0.081 (4)	−0.001 (3)	0.031 (3)	−0.009 (4)
C20	0.080 (5)	0.062 (5)	0.084 (5)	0.008 (4)	0.028 (4)	0.006 (4)
C21	0.067 (4)	0.064 (5)	0.098 (5)	−0.006 (4)	0.022 (4)	−0.018 (5)
C22	0.066 (4)	0.086 (6)	0.074 (4)	0.015 (5)	0.031 (3)	−0.003 (5)
C23	0.080 (4)	0.079 (5)	0.074 (5)	0.002 (5)	0.030 (4)	0.011 (4)
C24	0.076 (4)	0.069 (5)	0.085 (5)	0.001 (4)	0.030 (4)	−0.001 (4)

### Geometric parameters (Å, º)

**Table:**

Br1—C22	1.897 (6)	C10—C11	1.360 (10)
S1—C3	1.719 (7)	C10—H10	0.9300
S1—C1	1.720 (6)	C11—C12	1.388 (9)
N1—C3	1.295 (7)	C11—H11	0.9300
N1—C2	1.411 (7)	C12—H12	0.9300
N3—C6	1.290 (8)	C13—C18	1.384 (9)
N3—N2	1.373 (6)	C13—C14	1.386 (8)
F1—C16	1.365 (7)	C14—C15	1.395 (8)
N2—C3	1.378 (7)	C14—H14	0.9300
N2—C4	1.483 (8)	C15—C16	1.344 (9)
C1—C2	1.357 (8)	C15—H15	0.9300
C1—H1	0.9300	C16—C17	1.364 (10)
C2—C7	1.464 (8)	C17—C18	1.382 (8)
C4—C13	1.508 (7)	C17—H17	0.9300
C4—C5	1.531 (8)	C18—H18	0.9300
C4—H4	0.9800	C19—C24	1.388 (10)
C5—C6	1.493 (8)	C19—C20	1.383 (9)
C5—H5A	0.9700	C20—C21	1.377 (9)
C5—H5B	0.9700	C20—H20	0.9300
C6—C19	1.472 (9)	C21—C22	1.361 (10)
C7—C12	1.392 (8)	C21—H21	0.9300
C7—C8	1.392 (10)	C22—C23	1.379 (11)
C8—C9	1.377 (9)	C23—C24	1.394 (8)
C8—H8	0.9300	C23—H23	0.9300
C9—C10	1.358 (10)	C24—H24	0.9300
C9—H9	0.9300		
			
C3—S1—C1	87.6 (3)	C10—C11—C12	119.5 (8)
C3—N1—C2	108.7 (5)	C10—C11—H11	120.3
C6—N3—N2	108.5 (6)	C12—C11—H11	120.3
N3—N2—C3	118.9 (5)	C11—C12—C7	122.1 (7)
N3—N2—C4	113.8 (5)	C11—C12—H12	118.9
C3—N2—C4	124.1 (6)	C7—C12—H12	118.9
C2—C1—S1	111.7 (5)	C18—C13—C14	119.2 (5)
C2—C1—H1	124.1	C18—C13—C4	121.2 (6)
S1—C1—H1	124.1	C14—C13—C4	119.4 (6)
C1—C2—N1	114.2 (6)	C13—C14—C15	120.3 (6)
C1—C2—C7	128.2 (6)	C13—C14—H14	119.9
N1—C2—C7	117.6 (6)	C15—C14—H14	119.9
N1—C3—N2	121.5 (6)	C16—C15—C14	118.4 (6)
N1—C3—S1	117.8 (5)	C16—C15—H15	120.8
N2—C3—S1	120.6 (5)	C14—C15—H15	120.8
N2—C4—C13	110.7 (5)	C15—C16—F1	118.7 (7)
N2—C4—C5	100.2 (5)	C15—C16—C17	123.3 (6)
C13—C4—C5	114.7 (5)	F1—C16—C17	118.0 (7)
N2—C4—H4	110.3	C16—C17—C18	118.5 (7)
C13—C4—H4	110.3	C16—C17—H17	120.8
C5—C4—H4	110.3	C18—C17—H17	120.8
C6—C5—C4	104.1 (5)	C13—C18—C17	120.4 (7)
C6—C5—H5A	110.9	C13—C18—H18	119.8
C4—C5—H5A	110.9	C17—C18—H18	119.8
C6—C5—H5B	110.9	C24—C19—C20	117.9 (6)
C4—C5—H5B	110.9	C24—C19—C6	121.0 (6)
H5A—C5—H5B	108.9	C20—C19—C6	121.0 (6)
N3—C6—C19	120.8 (6)	C21—C20—C19	121.5 (7)
N3—C6—C5	113.0 (5)	C21—C20—H20	119.2
C19—C6—C5	126.1 (6)	C19—C20—H20	119.2
C12—C7—C8	116.5 (6)	C22—C21—C20	119.6 (7)
C12—C7—C2	120.8 (7)	C22—C21—H21	120.2
C8—C7—C2	122.6 (6)	C20—C21—H21	120.2
C9—C8—C7	120.7 (7)	C21—C22—C23	121.2 (6)
C9—C8—H8	119.7	C21—C22—Br1	119.4 (6)
C7—C8—H8	119.7	C23—C22—Br1	119.5 (6)
C10—C9—C8	121.5 (8)	C22—C23—C24	118.7 (7)
C10—C9—H9	119.2	C22—C23—H23	120.6
C8—C9—H9	119.2	C24—C23—H23	120.6
C9—C10—C11	119.7 (8)	C19—C24—C23	121.0 (7)
C9—C10—H10	120.1	C19—C24—H24	119.5
C11—C10—H10	120.1	C23—C24—H24	119.5
			
C6—N3—N2—C3	−158.5 (6)	C9—C10—C11—C12	−0.3 (11)
C6—N3—N2—C4	2.0 (7)	C10—C11—C12—C7	−0.1 (11)
C3—S1—C1—C2	−1.3 (5)	C8—C7—C12—C11	0.4 (10)
S1—C1—C2—N1	1.2 (7)	C2—C7—C12—C11	−177.8 (6)
S1—C1—C2—C7	179.6 (5)	N2—C4—C13—C18	−42.9 (8)
C3—N1—C2—C1	−0.3 (7)	C5—C4—C13—C18	69.5 (8)
C3—N1—C2—C7	−178.9 (5)	N2—C4—C13—C14	143.0 (6)
C2—N1—C3—N2	179.6 (5)	C5—C4—C13—C14	−104.5 (6)
C2—N1—C3—S1	−0.7 (7)	C18—C13—C14—C15	1.0 (9)
N3—N2—C3—N1	169.5 (6)	C4—C13—C14—C15	175.2 (6)
C4—N2—C3—N1	11.1 (9)	C13—C14—C15—C16	−0.6 (9)
N3—N2—C3—S1	−10.2 (8)	C14—C15—C16—F1	178.3 (6)
C4—N2—C3—S1	−168.5 (4)	C14—C15—C16—C17	0.8 (11)
C1—S1—C3—N1	1.2 (5)	C15—C16—C17—C18	−1.5 (10)
C1—S1—C3—N2	−179.1 (6)	F1—C16—C17—C18	−179.0 (6)
N3—N2—C4—C13	116.3 (5)	C14—C13—C18—C17	−1.6 (9)
C3—N2—C4—C13	−84.3 (7)	C4—C13—C18—C17	−175.7 (6)
N3—N2—C4—C5	−5.1 (6)	C16—C17—C18—C13	1.9 (9)
C3—N2—C4—C5	154.3 (6)	N3—C6—C19—C24	−179.8 (6)
N2—C4—C5—C6	5.8 (6)	C5—C6—C19—C24	−2.2 (10)
C13—C4—C5—C6	−112.8 (6)	N3—C6—C19—C20	−2.1 (9)
N2—N3—C6—C19	−179.7 (5)	C5—C6—C19—C20	175.5 (6)
N2—N3—C6—C5	2.4 (7)	C24—C19—C20—C21	2.5 (10)
C4—C5—C6—N3	−5.5 (7)	C6—C19—C20—C21	−175.2 (6)
C4—C5—C6—C19	176.7 (6)	C19—C20—C21—C22	−0.4 (10)
C1—C2—C7—C12	−10.0 (10)	C20—C21—C22—C23	−1.8 (11)
N1—C2—C7—C12	168.3 (6)	C20—C21—C22—Br1	176.9 (5)
C1—C2—C7—C8	172.0 (6)	C21—C22—C23—C24	1.8 (10)
N1—C2—C7—C8	−9.7 (9)	Br1—C22—C23—C24	−176.9 (5)
C12—C7—C8—C9	−0.3 (10)	C20—C19—C24—C23	−2.5 (10)
C2—C7—C8—C9	177.8 (6)	C6—C19—C24—C23	175.2 (6)
C7—C8—C9—C10	−0.1 (11)	C22—C23—C24—C19	0.4 (10)
C8—C9—C10—C11	0.4 (12)		

### Hydrogen-bond geometry (Å, º)

Cg1 is the centroid of the C13–C18 benzene ring.

**Table:**

*D*—H···*A*	*D*—H	H···*A*	*D*···*A*	*D*—H···*A*
C4—H4···S1^i^	0.98	2.84	3.734 (7)	153
C22—Br1···*Cg*1^ii^	1.90 (1)	3.64 (1)	5.265 (7)	142 (1)

Symmetry codes: (i) *x*, *y*−1, *z*; (ii) −*x*+1, *y*+1/2, −*z*+2.
